# The Graduating European Dentist Curriculum Framework: A 7‐Year Review

**DOI:** 10.1111/eje.13058

**Published:** 2024-11-20

**Authors:** James Field, Sibylle Vital, Jonathan Dixon, Denis Murphy, Julia Davies

**Affiliations:** ^1^ Cardiff University Cardiff UK; ^2^ UFR Odontology, AP‐HP Universite Paris Cite Paris France; ^3^ The University of Sheffield Sheffield UK; ^4^ ADEE Dublin Ireland; ^5^ Malmo University Malmo Sweden

**Keywords:** curriculum, dental, education, oral health

## 1 Introduction and Background

By its very nature, the Association for Dental Education in Europe (ADEE) is an international voice of dental education, representing dental schools throughout Europe since 1975. The concept of converging and harmonising Oral Health Professional (OHP) education across Europe is at the heart of ADEE's mission and has led to the publication of a number of consensus documents that have become recognised benchmarks for institutions and regulatory bodies across the world. ADEE is also ideally positioned, through regional representation, to engage with multiple European and wider global stakeholders. The pan‐European taskforce approach used by ADEE is supported by the recognition that similar social partnerships (involving a wide range of stakeholders) have been shown to increase the validity of curricula, facilitate the transition of students to a vocational work environment, and to help students develop into proficient and effective practitioners [1, 2].

Around 25 years ago, a successful European Union‐funded Thematic Network Project (DentEd) commenced, which initially aimed to facilitate the convergence of Dental Education across Europe. The initiative eventually ran over a 9‐year period as three interlinked projects:

- the profiles and competences of a graduating European dentist
- the curriculum structure, content, learning and assessment
- quality assurance and benchmarking

Whilst the Profiles and Competences project, which provided a profile of the competences that a newly graduated dentist should be able to demonstrate, was the most popular in isolation, all three publications have proved instrumental in shaping the delivery of dental education across Europe [3, 4, 5]. The popularity and influence of the DentEd project were demonstrated by the number of downloads and citations that have taken place, and the documents have been used by many schools, educational establishments and professional organisations globally, to support the development and/or benchmarking of undergraduate dental curricula.

Due to the broad interpretation of ‘competences’, authors, organisations, institutions, regulators and societies gradually shifted towards a ‘Learning Outcomes’ approach. As such, in 2015 a new taskforce was established to revisit, reconsider and accordingly revise the content, and the ideologies, that should underpin a modern European dental curriculum.

In 2017, a new suite of five curriculum papers was published, collectively titled The Graduating European Dentist (GED). The initial introductory paper provided context and rationale for the learning‐outcomes‐based approach. This paper is, at the time of publication, the third most‐cited paper within the European Journal of Dental Education (EJDE). Four of the papers mirrored the new curriculum domains; and in turn, each was defined by areas of ‘Major Competence’ (Appendix) and provided a basis from which graduates could build confidence and competence towards becoming independent practitioners, who accept the importance of continuing professional development throughout their career. The paper that focuses on methods of Teaching, Learning and Assessment is, at the time of print, the sixth most‐accessed paper within the *European Journal of Dental Education*. The papers, and their citations, are listed in Table 1.

**TABLE 1:** Papers, links, and citations for the Graduating European Dentist curriculum.

Paper title	DOI	Reference	Citations at time of print
Commentary and introductory paper	10.1111/eje.12307	[6]	201
1: Professionalism	10.1111/eje.12308	[7]	36
II: Safe and Effective Clinical Practice	10.1111/eje.12309	[8]	32
III: Patient‐Centred Care	10.1111/eje.12310	[9]	43
IV: Dentistry in Society	10.1111/eje.12311	[10]	24
V: Research	10.1111/eje.13040	[11]	0 (*new*)
Methods of teaching and assessment	10.1111/eje.12312	[12]	72

*Note:* Citation information provided by Google Scholar.

### 1.1 GED Impact

The new GED framework has proven very popular with educators, as demonstrated by the fact that the documents themselves have been cited over 400 times. They are emerging as a key reference for discussing the expectations of graduate dentists across Europe. The new approach, based on learning outcomes, is helping to facilitate better pedagogical alignment with local curricula.

In 2020, the Irish Dental Council adopted the GED as a basis for its own regulatory framework—and recent data collection across Europe through the Erasmus‐funded O‐Health‐Edu project has shown that the GED is utilised locally by almost 60% of responding schools (Dixon et al. [20, 21]). This demonstrates the positive impact that GED is having on a local‐level with individual institutions. Moreover, in November 2023, the Federation of European Dental Competent Authorities and Regulators (FEDCAR) endorsed the use of the GED curriculum nationally to its European members, including candidate countries [13]. In doing so, FEDCAR welcomed the GED's relevance to the European dental education context and highlighted the consensus and collaborative approach taken by ADEE in creating the curriculum.

ADEE's stakeholder approach meant that the GED documents were also utilised by the European Dental Hygienists Federation, which represents 24 member countries and over 38 000 dental hygienists, to develop its own Common Education Curriculum (CEC) in 2020—the first of its kind, supported by ADEE. These curriculum documents (Table 2) have also been popular, with over 40 citations to date.

**TABLE 2:** Papers, links, and citations for the Common Education Curriculum for Dental Hygiene.

Paper title	DOI	Reference	Citations at time of print
Commentary and introductory paper	10.1111/eje.12511	[14]	23
1: Professionalism	10.1111/eje.12508	[15]	4
II: Safe and Effective Clinical Practice	10.1111/eje.12509	[16]	6
III: Patient‐Centred Care	10.1111/eje.12510	[17]	10
IV: Oral Health in Society	10.1111/eje.12511	[18]	4

*Note:* Citation information provided by Google Scholar.

In terms of wider representation, ADEE was instrumental in bringing together nine trans‐European partner schools as part of an EU‐funded collaborative Erasmus + project (O‐Health‐Edu) that commenced in 2019. The overarching aims of the project were to better understand the existing state of Oral Health Professional (OHP) education in Europe and to develop a common vision for education, with the GED framework at its heart [19]. The findings from this project have now been published as open‐access journal articles [20, 21]. The project also saw the production and publication of several key academic resources—most notably a Vision for OHP education [22], an online glossary of educational terms, ‘Articulate’, to harmonise understanding of terminology across Europe, [23] and a scoping review of current educational practice across Europe [24].

### 1.2 GED Dissemination

In early 2020, ADEE developed a dedicated website to publish a digital version of the GED curriculum. Following a rolling programme of feedback, the digital resource was revisited in 2023 to offer a more modern feel, with increased functionality. The new GED pages (https://adee.org/graduating‐european‐dentist) provide easy access to the curriculum learning outcomes—but also offer new easy‐to‐access information about the GED taskforce itself including its membership, and its recent activities and updates.

**FIGURE 1:**
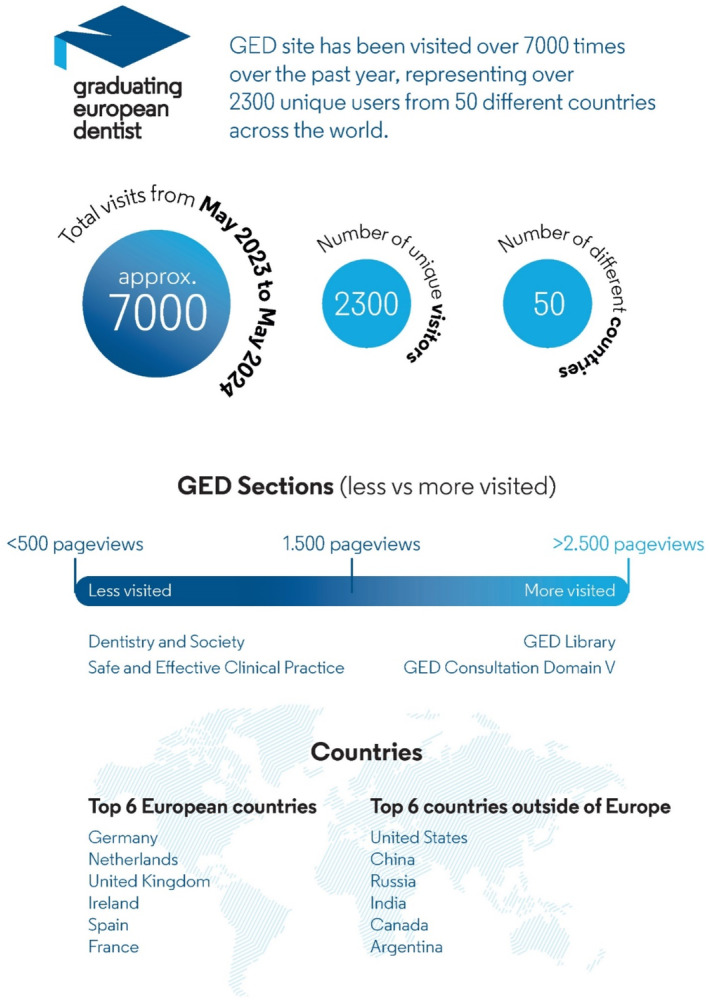
Data for the Graduating European Dentist pages.

From May 2023 to May 2024, the GED site has been visited over 7000 times, representing over 2300 unique visitors from over 50 different countries. This demonstrates the increasing awareness of the GED site and its accessibility to users across the world. The website receives on average 250 visitors per month, with the GED curriculum library and the pages relating to the online consultations, being the most frequented. The top European country for access was Germany, and outside of Europe was the United States. It is difficult at this stage to rationalise why certain countries show a significantly higher visitation rate—although given the collaborative nature of ADEE and ADEA (American Dental Education Association) it might not be surprising that there is significant interest from the United States. These statistics and more are represented in Figure 1.

**FIGURE 2:**
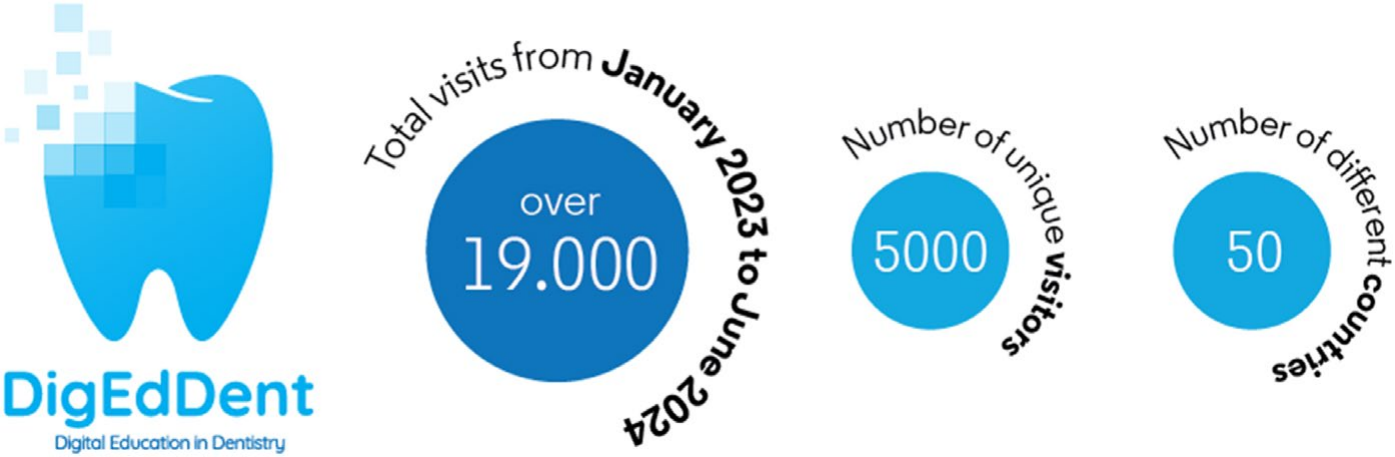
Data for DigEdDent site.

Prior to the COVID‐19 pandemic, the GED taskforce had been working on an online resource for supporting the digital delivery of dental education. The ‘DigEdDent’ project (https://adee.org/digeddent‐digital‐education‐dentistry) draws together resources across a number of domains, to help educators teach, assess, communicate, support and track students as we progress further into the digital age. This part of the site has also proved to be very popular. Between January 2023 and January 2024, the DigEdDent site was visited over 19 000 times by over 5000 unique visitors, from over 50 different countries. There are an average of 900 visitors per month—and the most‐visited sections of the site are those relating to digital student support and digital communications. These statistics are represented in Figure 2.

This increased visibility and utilisation of the ADEE site, as the ‘home’ of the GED, has seen various European organisations and institutions approach ADEE to help with their own curriculum reviews. As such, the taskforce has offered its expertise—but also utilised the online platform to host innovative Delphi‐style surveys to support the process. Examples of partnerships for curriculum development include the British Prosthodontic Society, the European Federation of Periodontology, and the European Endodontic Society.

### 1.3 GED Developments

Keeping pace with changes in our profession, training and society, two significant updates to the GED curriculum were developed: learning outcomes in relation to Environmental Sustainability [25] and Research [11]. Both processes, which included a process of open consultation, considered the development of behaviour‐based learning outcomes in relation to the new GED curriculum domains. As such, the taskforce was able to implement and highlight the changes easily online.

As part of the web resources, the GED ‘In Focus’ and ‘Updates’ sections provide regular accessible updates by members of the taskforce around contemporary areas of interest or importance. A series of podcasts is also provided to update interested users on the activities of the group. Web traffic data shows that after sending ADEE newsletters or posting these updates on social media, engagement with the site increases by over 20% in the following 3–5 days.

Most recently, a curriculum library was developed, which acts as a repository for relevant published papers that represent curriculum content, current practice, or methods of teaching and assessment. This library is regularly updated by the GED taskforce and can be easily filtered or searched for relevant materials. Examples of current content are provided in Table 3.

**TABLE 3:** Most recent key papers included in the current curriculum library—which can be found within the ADEE Graduating European Dentist pages at: https://adee.org/graduating‐european‐dentist/graduating‐european‐dentist‐curriculum.

*Anatomy*	10.1111/joa.13133	The Anatomical Society's Core Anatomy Syllabus for Dental Undergraduates [26]
*Artificial Intelligence*	10.1016/j.jdent.2022.104363	Artificial Intelligence for Oral and Dental Healthcare: Core Education Curriculum [27]
*Biological and Biomedical Sciences*	10.1111/eje.12518	A Core Curriculum in the Biological and Biomedical Sciences for Dentistry [28]
*Cariology*	10.1159/000330006	European Core Curriculum in Cariology for Undergraduate Dental Students [29]
*Dental Trauma*	10.1111/edt.12906	International Teaching Practices in Dental Trauma Education [30]
*Endodontics*	10.1111/iej.14064	European Society of Endodontology—Undergraduate Curriculum Guidelines for Endodontology [31]
*Environmental Sustainability*	10.1016/j.jdent.2024.105021	Curriculum Content for Environmental Sustainability in Dentistry [32]
	10.1111/eje.12852	Embedding Environmental Sustainability Within Oral Health Professional Curricula—Recommendations for Teaching and Assessment of Learning Outcomes [25]
	10.1111/eje.12631	Embedding Environmental Sustainability Within the Modern Dental Curriculum— Exploring Current Practice and Developing a Shared Understanding [33]
	10.1111/eje.13033	Environmental Sustainability in Oral Health Professional Education: Approaches, Challenges, and Drivers—ADEE Special‐Interest Group Report [34]
*Food Science and Nutrition*	10.1111/eje.12822	Implementation of a Food Science and Nutrition Module in a Dental Undergraduate Curriculum [35]
*General Curricula*	10.1111/eje.12989 10.1111/eje.12987	O‐Health‐Edu: A Viewpoint Into the Current State of Oral Health Professional Education in Europe: Part 1 and Part II [20, 21]
*Gerodontology*	10.1111/j.1741‐2358.2009.00296.x	European College of Gerodontology: Undergraduate Curriculum Guidelines in Gerodontology [36]
*Implant Dentistry*	10.1111/j.1600‐0579.2008.00556.x	Teaching and Assessment of Implant Dentistry in Undergraduate and Postgraduate Education: A European Consensus [37]
*Operative Skills*	10.1111/eje.12595	Defining Dental Operative Skills Curricula: An ADEE Consensus Paper [38]
	10.1111/eje.12276	Curriculum Content and Assessment of Pre‐Clinical Dental Skills: A Survey of Undergraduate Dental Education in Europe [39]
*Oral Pathology and Oral Medicine*	10.1111/j.1600‐0579.2012.00758.x	Scandinavian Fellowship for Oral Pathology and Oral Medicine: Guidelines for Oral Pathology and Oral Medicine in the Dental Curriculum [40]
	10.1111/eje.12366	Oral Medicine for Undergraduate Dental Students in the United Kingdom and Ireland—A Curriculum [41]
*Orthodontics*	10.1093/ejo/cjt059	The Erasmus Programme for Postgraduate Education in Orthodontics in Europe: an Update of the Guidelines [42]
*Periodontology*	10.1111/jcpe.13991	Domains, Competences and Learning Outcomes for Undergraduate Education in Periodontology [43]
*Prosthodontics*	10.1016/j.jdent.2022.104207	Undergraduate Teaching and Assessment Methods in Prosthodontics Curriculum: An International Delphi Survey [44]
*Special Care*	10.1111/eje.12054	Guidance for the Core Content of a Curriculum in Special Care Dentistry at the Undergraduate Level [45]

### 1.4 Future Work

Following strategic direction from the ADEE executive board, the GED taskforce will now embark on a process of pan‐European consultation to take a deeper dive into the uptake and use of the GED curriculum framework. Building on the work by Dixon et al. [20, 21], the project will seek to better understand the nuances of how the GED framework is able to be implemented at a local level. As a result, it is hoped that the actions of the GED taskforce will be channelled towards necessary and impactful activities that result in further useful resources for the academic community.

## Conflicts of Interest

The authors declare no conflicts of interest.

## Data Availability

The authors have nothing to report.

## GED Domains and Their Major Competences

Domain I: Professionalism

– 1.1 Ethics
– 1.2 Regulation
– 1.3 Professionalism

Domain II: Safe and effective clinical practice

– 2.1 Evidence‐based Practice
– 2.2 Management and Leadership
– 2.3 Team‐working and Communication
– 2.4 Audit and Risk Management
– 2.5 Professional Education and Training

Domain III: Patient‐centred Care

– 3.1 Applying the Scientific Basis of Oral Healthcare
– 3.2 Clinical Information Gathering and Diagnosis
– 3.3 Treatment Planning
– 3.4 Establishing and Maintaining Oral Health

Domain IV: Dentistry in Society

– 4.1 Dental Public Health
– 4.2 Prevention and Health Promotion
– 4.3 Inter‐Professional Collaboration
– 4.4 Health Advocacy

Domain V: Research

– 5.1 Research Design
– 5.2 Data Analysis and Interpretation
– 5.3 Information Literacy
